# An unexpected guest

**DOI:** 10.11604/pamj.2017.27.140.13022

**Published:** 2017-06-28

**Authors:** Amina Kissou, Badr Eddine Hassam

**Affiliations:** 1 IBN SINA University Hospital, Rabat, Morocco

**Keywords:** Lesion, amlodipine, papillomatosis, acanthosis

## Image in medicine

A 60-year-old woman, diabetic with amlodipine, consulted for a rapidly progressive lesion in the back of the hand. The examination found a hard, sessile, painful lesion with purple-colored and keratosic surface. It was 1cm in diameter. Total removal of the lesion with 2mm margin was indicated. The histological examination showed the presence of papillomatosis and acanthosis with koilocytes and the diagnosis of wart was retained. The clinical presentation of this lesion is a typical; it is a lesion on the dorsal margin of the hand, painful, purple-colored, with a rapid evolution in 01mois. A keratoacanthoma or epidermoid carcinomas were both suspected firstly. After resection there was no recurrence with a follow-up of 4 years.

**Figure 1:**
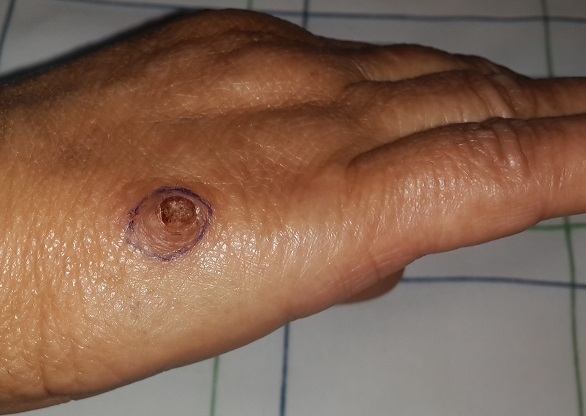
Sessile lesion on the hand

